# Seedless Cu Electroplating on Co-W Thin Films in Low pH Electrolyte: Early Stages of Formation

**DOI:** 10.3390/nano11081914

**Published:** 2021-07-25

**Authors:** Rúben F. Santos, Bruno M. C. Oliveira, Alexandre Chícharo, Pedro Alpuim, Paulo J. Ferreira, Sónia Simões, Filomena Viana, Manuel F. Vieira

**Affiliations:** 1 Department of Metallurgical and Materials Engineering, University of Porto, Rua Dr. Roberto Frias, 4200-465 Porto, Portugal; up200803851@edu.fe.up.pt (B.M.C.O.); ssimoes@fe.up.pt (S.S.); fviana@fe.up.pt (F.V.); mvieira@fe.up.pt (M.F.V.); 2 LAETA/INEGI–Institute of Science and Innovation in Mechanical and Industrial Engineering, Rua Dr. Roberto Frias, 4200-465 Porto, Portugal; 3 International Iberian Nanotechnology Laboratory, Av. Mestre José Veiga, 4715-330 Braga, Portugal; alexandre.chicharo@inl.int (A.C.); pedro.alpuim.us@inl.int (P.A.); paulo.ferreira@inl.int (P.J.F.); 4 Centre of Physics, University of Minho, 4710-057 Braga, Portugal; 5 Materials Science and Engineering Program, University of Texas at Austin, Austin, TX 78712, USA; 6 Mechanical Engineering Department and IDMEC, IST University of Lisbon, Av. Rovisco Pais, 1049-001 Lisboa, Portugal

**Keywords:** seedless electroplating, Cu, Co-W, interconnect, acidic

## Abstract

The use of Ta/TaN barrier bilayer systems in electronic applications has been ubiquitous over the last decade. Alternative materials such as Co-W or Ru-W alloys have gathered interest as possible replacements due to their conjugation of favourable electrical properties and barrier layer efficiency at reduced thicknesses while enabling seedless Cu electroplating. The microstructure, morphology, and electrical properties of Cu films directly electrodeposited onto Co-W or Ru-W are important to assess, concomitant with their ability to withstand the electroplating baths/conditions. This work investigates the effects of the current application method and pH value of the electroplating solution on the electrocrystallisation behaviour of Cu deposited onto a Co-W barrier layer. The film structure, morphology, and chemical composition were studied by X-ray diffraction, scanning electron microscopy and atomic force microscopy, as well as photoelectron spectroscopy. The results show that the electrolyte solution at pH 1.8 is incapable of creating a compact Cu film over the Co-W layer in either pulsed or direct-current modes. At higher pH, a continuous film is formed. A mechanism is proposed for the nucleation and growth of Cu on Co-W, where a balance between Cu nucleation, growth, and preferential Co dissolution dictates the substrate area coverage and compactness of the electrodeposited films.

## 1. Introduction

Following the trend established by Moore’s Law, more transistors are being packed in a single chip as the dimensions continue to downscale. As of 2020, the transistor count is on the order of several tens of billions in CPU and GPU systems. This continuous miniaturisation makes the massive spread of electronic devices, computers, smartphones, and wearables possible, which is also followed by great manufacturing challenges, where conventional processes and materials are reaching their usability limits and thus requiring either an improvement or replacement. In this regard, copper interconnects, which act as electrical paths in integrated circuits, have also been downscaled, thereby experiencing a reduction in width and thickness. Yet, the process of metallisation of these copper interconnects requires a diffusion barrier layer on the sidewalls/bottoms of vias and lines to prevent Cu atoms from diffusing into the surrounding dielectrics. Currently, a TaN diffusion barrier layer and a Ta adhesion layer are employed to improve the bonding between Cu and TaN. Although Ta/TaN is effective as a diffusion barrier system, it increases the global interconnect resistivity as the miniaturisation progresses further, ultimately limiting the performance of the integrated circuits. Furthermore, Cu dewetting and electromigration—phenomena related to the adhesion strength between Cu and Ta [1,2]—are becoming more prominent, hence limiting the interconnect reliability [3,4]. In addition, Ta/TaN requires a Cu seed layer on top before the electrolytic deposition process is initiated in order to enable adequate via and line filling. Uniform Cu-seed/Ta/TaN layers have thus become increasingly more difficult to deposit over increasingly narrow vias using conventional fabrication techniques.

Alternative barrier layers have been considered to replace Ta/TaN, such as Ru- and Co-based binary systems—e.g., Ru-W, Ru-Mn, and Co-W [5,6,7]. The Co-W system is an interesting candidate as a novel barrier material due to its electrical and thermal properties. Co exhibits a relatively low electrical resistivity in a wide range of thicknesses [8,9,10], whereas W enhances the diffusion barrier properties [11], preventing Cu from diffusing into the dielectrics. In [11], it was shown that CVD-Co-W (with 20 at% W) displays equivalent diffusion barrier properties to PVD-TaN. Additionally, the interface adhesion strength in Cu/Co-W is higher than in Cu/Ta [12,13], favouring lower Cu diffusion along the interface Cu/barrier and subsequently improving the electromigration lifetime [14]. From a manufacturing point of view, Co-W is an interesting alternative to Ta/TaN as well, because it can be synthesised by electrochemical routes that produce a high film uniformity and step coverage in narrow vias and lines. Additionally, Co-W can function as both a diffusion barrier and seed layer, whereupon Cu can be directly electroplated [15]. Electroless methods used for the synthesis of Co-W-based thin films have been reported in the literature [16,17,18], but the direct electroplating of Cu on Co-W-based thin films is frankly unaddressed. The feasibility of Co-W as a directly electroplatable diffusion barrier layer to interconnect metallisation depends on its capability to grow a Cu film on top, with adequate morphological and microstructural characteristics and equivalent or superior electronic performance to conventional barrier layer systems. This work proposes a method for Cu electrocrystallisation on top of Co-W thin films using conventional acidic electroplating baths, focusing on the initial stages of Cu film formation.

## 2. Experimental

### 2.1. Co-W Thin Film Deposition

A ≈ 100 nm layer of SiO_2_ was deposited on top of a p-type boron-doped Si (100) wafer (Silicon Valley Microelectronics, Santa Clara, CA, USA) by plasma-enhanced chemical vapour deposition (PECVD) with a high radio frequency in a CVD MPX chamber (SPTS Technologies Ltd., Newport, UK). The wafer was diced into 15 mm × 15 mm substrates, whereupon Co-W films were deposited by DC magnetron sputtering in a confocal ultra-high vacuum sputtering system (Kenosistec, Binasco, Italy). Co and W were simultaneously sputtered from their respective targets (99.95%, Testbourne Ltd., Basingstoke, UK) for 600 s. Films with different W contents were deposited by applying a power bias between 40 and 100 W on the W target while keeping the Co target fixed at 40 W. The base and working pressures were 6.4 × 10^−5^ and 6.9 × 10^−1^ Pa, respectively, maintaining an Ar flux of 20 sccm into the sputtering chamber. Co-W film thickness was measured using a contact profilometry instrument (KLA-Tencor, Milpitas, CA, USA).

### 2.2. Cu Electroplating

Cu was directly electroplated on Co-W/SiO_2_/Si substrates using an acidic electrolyte of 0.05 M CuSO_4_∙5H_2_O (99.995%, Sigma-Aldrich, St. Louis, MO, USA), 0.05 M H_2_SO_4_ (Honeywell/Fluka, Charlotte, NC, USA), 1 mM NaCl (Honeywell/Fluka, Charlotte, NC, USA), and 300 ppm polyethylene glycol (PEG) 600 (Fluka Chemie GmbH, Buchs, Switzerland) in double-distilled water. Electrolyte acidity was measured with a FiveEasy™ F20 digital pH meter (Mettler Toledo, Greifensee, Switzerland). Galvanostatic direct current (DC) and pulsed current (PC) plating routines were employed using a Gamry potentiostat/galvanostat Interface 1000E (Gamry Instruments, Warminster, PA, USA) with a simple two-electrode cell configuration, removing the need for the use of reference electrodes. The plating was carried out at room temperature in a 200 cm^3^ poly(methyl methacrylate) container without electrolyte stirring using a >99.9% pure Cu coil as the counter-electrode (anode) fixed at a ≈15 mm distance from the substrate (cathode). Prior to each plating routine, the substrates were pre-treated in a 1:20 H_2_SO_4_ solution for 60 s to remove possible oxidation present on the Co-W film surface, followed by rinsing in double-distilled water for another 60 s, both under ultrasonic agitation. The substrates were then dried by Ar blowing and masked with a polymeric film, limiting the exposed area to a circle of 0.20 cm^2^. After deposition, substrates were immediately removed from the electrolyte, rinsed in double-distilled water, and dried with soft Ar blowing.

### 2.3. Structural and Chemical Characterisation of Substrate and Cu Films

The as-sputtered Co-W film surfaces were observed by scanning electron microscopy, SEM (Thermo Fisher Scientific Quanta 400FEG ESEM, Thermo Fisher Scientific, Hillsboro, OR, USA), and their composition was estimated by energy-dispersive X-ray spectroscopy, EDS (EDAX Genesis X4M, AMETEK, Berwyn, PA, USA). The compositional analysis was complemented by X-ray photoelectron spectroscopy, XPS (ESCALAB™ 250 Xi, Thermo Fisher Scientific, Waltham, MA, USA), and the film structure was determined by grazing incidence X-ray diffraction (GIXRD) at an angle of 1.5° using Cu Kα radiation (λ = 1.54060 Å) and a step size of 0.02 °·s^−1^. The surfaces of the Cu/Co-W films were also observed by SEM and optical microscopy (Leica DM4000 M, Leica Microsystems GmbH, Wetzlar, Germany), where the substrate–film interface cross-section was prepared by focused ion-beam milling (Thermo Fisher Scientific Helios 450S, Thermo Fisher Scientific, Waltham, MA, USA). Surface roughness changes in response to the chemical pre-treatment were measured by atomic force microscopy, AFM (Veeco Metrology Multimode, Veeco Instruments Inc., Oyster Bay, NY, USA), with a Bruker TESPA-V2 tip. Quantitative image analysis was employed on SEM images using the *ImageJ* software version 1.51p, National Institutes of Health, Bethesda, MD, USA.

## 3. Results and Discussion

**Figure 1:**
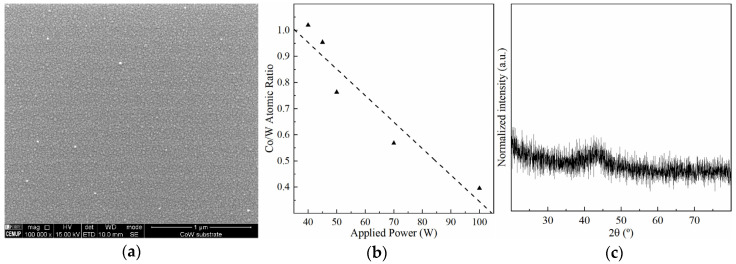
SEM image of Co-W thin film surface (**a**), Co/W atomic ratio as a function of W target power determined by EDS (**b**), and film grazing incidence X-ray diffractogram (**c**).

The sputtering conditions required to produce Co-W thin films with the desired chemical compositions were first investigated. As mentioned above, the power applied to the Co target was fixed at 40 W, while the one applied to the W target varied between 40 and 100 W. Sputtered films with 25 nm in thickness (measured by contact profilometry) display a uniform smooth surface (Figure 1a). Estimating the chemical composition of such Co-W thin films using EDS at 15 keV requires special attention due to the large interaction volume, in the range of micrometres, picking up a strong X-ray signal from the SiO_2_/Si underneath. EDS spectra were obtained with a 15 keV electron beam to excite Co K and W L spectral lines, which do not overlap with Si and O emission lines, and were used for elemental semi-quantification after 200 s of acquisition time to improve the signal-to-noise ratio. Higher Co/W atomic ratios were obtained as the power on the W target decreased (Figure 1b), but due to the inherent limitations mentioned before, these values should be considered more as a qualitative assessment. Co-W films near equimolar composition were selected in this work for subsequent Cu electrodeposition. Su et al. [15] demonstrated that equimolar Co-W films display the best plating behaviour while being effective as a diffusion barrier. GIXRD confirms the amorphous structure of the film, allowing it to adequately function as a diffusion barrier layer (Figure 1c).

**Figure 2:**
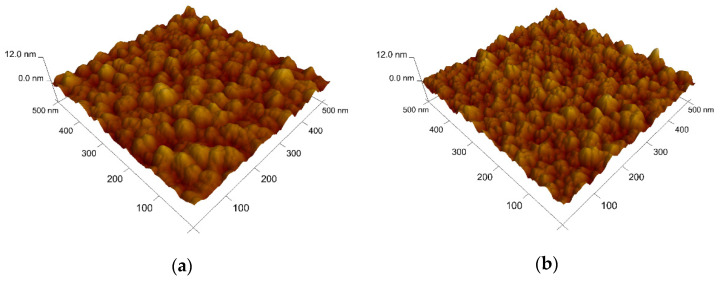
AFM images of the surface of the Co-W film before (**a**) and after (**b**) the sulphuric acid pre-treatment.

The effect of the sulphuric acid pre-treatment before electroplating was evaluated by AFM in tapping mode. By comparison fo the images in Figure 2, it can be seen that the pre-treatment etched the surface, leaving a slightly more sharpened profile. The average surface roughness (*S_a_*) of a 500 nm × 500 nm area was determined to be 1.01 nm for the untreated surface, and 1.05 nm after pre-treatment, using the *NanoScope* software 6.13R1 (Veeco Instruments Inc., Oyster Bay, NY, USA). Cu electrodeposition was conducted under a strict procedure to reduce the contact time between the substrate and the electrolyte to the minimum necessary for deposition, and to ensure maximum reproducibility between plating events. A comparison of the results of different plating conditions was undertaken using the central regions of the films. PC electrodeposition was employed as an alternative to DC for its added capability to not only control the applied current density, but also its on (*t_on_*) and off (*t_off_*) times. PC Cu films have been reported to display better electrical, thermal, and mechanical properties when compared to its DC counterparts [19,20,21]. A comparison between PC and DC electrodeposition modes can also provide further insight into the Cu nucleation and growth on Co-W thin films.

To isolate the effect of pulsed current density, *j_p_*, on Cu nucleation and growth, deposition times were decreased from 208 to 26 s with an increase in *j_p_* from 5 to 40 mA·cm^−^^2^, maintaining the total electrical charge supplied to the substrate unchanged. According to expression (1),

(1) $$Q=j_{p}\eta t,$$

where *Q* is the total charge supplied in C, *η* the duty cycle given by Equation (2) and *t* the total deposition time in s.

(2) $$\eta =\frac{t_{on}}{t_{on}+t_{off}}.$$

**Figure 3:**
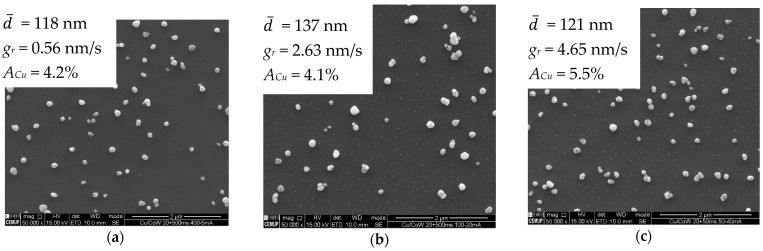
SEM images of Cu particles on Co-W substrate electrodeposited by PC at 5 (**a**), 20 (**b**) and 40 mA·cm^−2^ (**c**) for periods of 208, 52, and 26 s, respectively (constant *Q* = 40 mC, *t_on_* = 50 ms, and *t_off_* = 500 ms).

**Figure 4:**
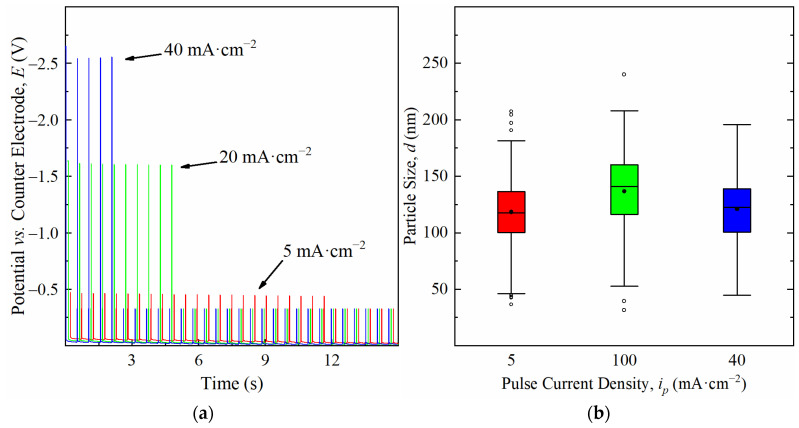
Chronopotentiometric curves of the first seconds of deposition (**a**) and box plots of the particle size (coarse particles only) (**b**) obtained from 5 to 40 mA·cm^−2^.

The Cu particles form on Co-W by instantaneous nucleation, whereby the nuclei density is higher when *j_p_* rises to 20 mA·cm^−^^2^ (Figure 3). The number of active sites for nucleation reaches saturation around this current density, since it does not increase for higher *j_p_*. Nucleation seems to occur during the first pulses of current, followed by nuclei growth, as soon as it becomes more energetically favourable, as indicated by the drop in cathodic potentials seen in the chronopotentiometric curves obtained for each deposition (Figure 4a). Only a relatively small number of nuclei grow, whereas the remaining preserve what appears to be an embryonic-state, with sizes of less than 20 nm, resulting in a bimodal particle size distribution. It is noteworthy that the number of growing particles is approximately the same, regardless of *j_p_*. PC deposition is reported to promote a faster growth of specific crystallographic orientations [22,23], which could in part explain the preferential growth of a few nuclei, due to their more favourable orientation. However, this does not explain why the number of growing particles at 20 mA·cm^−2^ is near the same as the one observed at 5 mA·cm^−2^. Such an effect results in particles reaching a similar average size and identical distribution profiles regardless of nuclei density (Figure 4b). This indicates that higher current densities increase particle growth rates but fail to promote better substrate coverage, which was confirmed by measuring the substrate area occupied by Cu particles, *A_Cu_*. The growth rate, *g_r_*, refers to the average velocity of 2D expansion of the particles given by Equation (3), where *Δ*$\bar{d}$ is the increase in average particle size and *Δt* the time required for growth. Values of d represent particle diameter and were determined from the 2D projected area, a, on the substrate according to Equation (4). A minimum of 200 coarse particles were measured per condition. Very small particles were not considered in the calculation of $\bar{d}$.

(3) $$g_{r}=\frac{\Delta \bar{d}}{\Delta t},$$

(4) $$d=2\sqrt{\frac{a}{\pi }}.$$

**Figure 5:**
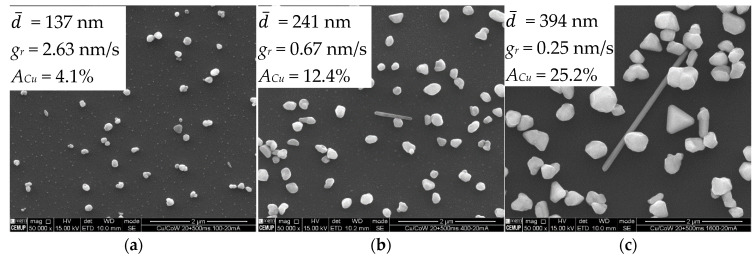

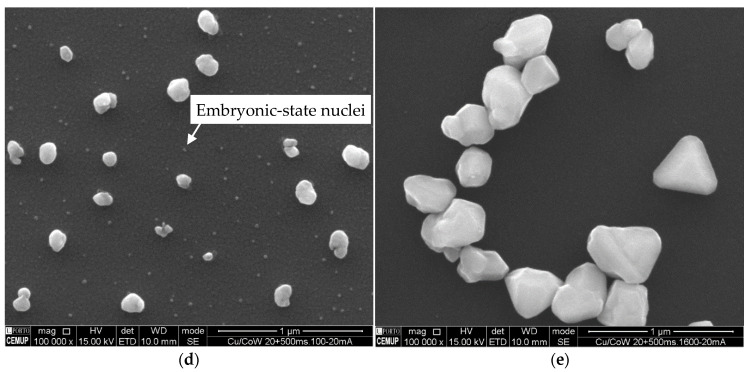
SEM images of Cu particles on Co-W substrate electrodeposited by PC at 20 mA·cm^−2^ for periods of 52 (**a**), 208 (**b**), and 832 s (**c**) (*t_on_* = 50 ms and *t_off_* = 500 ms). Higher magnifications of (**a**,**c**) in (**d**,**e**), respectively.

**Figure 6:**
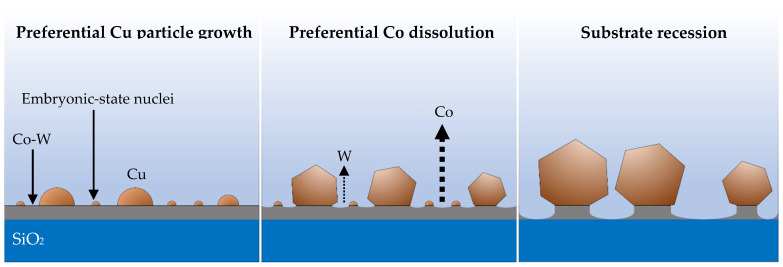
Mechanism and stages of Cu particle growth, on a dissolving Co-W substrate, rendering a discontinuous Cu film. Co dissolves faster than W.

Longer deposition times (at constant *j_p_*) promote further growth at lower rates (Figure 5). Particles increase in volume through a preferential crystallographic growth mechanism, leading to more polygonal/faceted shapes (Figure 5a–c). The embryonic-state nuclei that did not grow after 52 s of deposition (Figure 5d), remained mostly unaltered after 208 s, becoming undetectable in SEM imaging after 832 s (Figure 5e). These nuclei disappear either by direct dissolution into the electrolyte or by dissolution of the substrate underneath them. The acidic nature of the electrolyte herein used (pH ≈ 1.8) can dissolve Cu but is particularly aggressive to the Co-W substrate [15,24]. The nucleation and growth mechanism for Cu and the effects of the substrate dissolution are illustrated in Figure 6.

**Figure 7:**
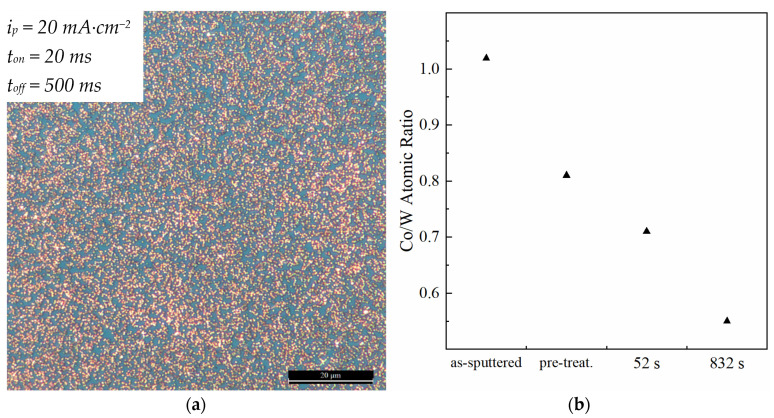
Same condition as Figure 5c observed by optical microscopy. Substrate dissolution reveals blue hue of SiO_2_ layer underneath it (**a**). Co/W atomic ratio before/after pre-treatment, and after different immersion times in electrodeposition electrolyte, obtained by EDS (**b**).

**Figure 8:**
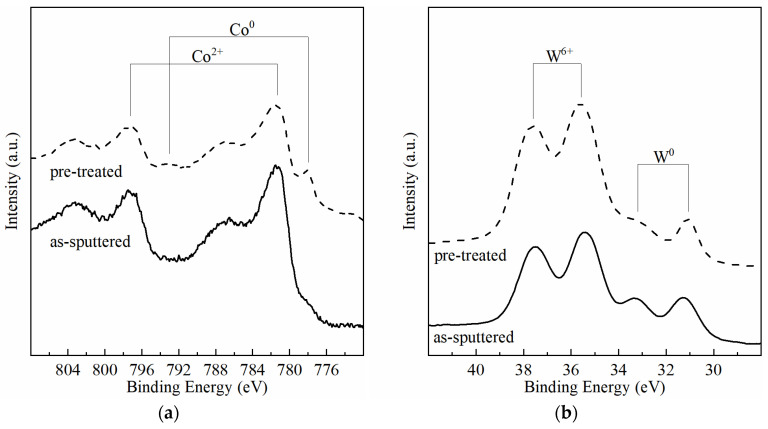
XPS spectra of elemental Co (**a**) and W (**b**) in the as-sputtered and pre-treated conditions.

The incorporation of W in the Co film reduces the substrate dissolution rate but does not prevent it. After 832 s of deposition, the SiO_2_ layer becomes visible in optical microscopy, confirming the dissolution/recession of the Co-W substrate (Figure 7a). EDS analysis reveals a decrease in the Co/W atomic ratio with increasing immersion times (Figure 7b), indicating that substrate recession is mediated by a quicker dissolution of Co. XPS analysis detects the presence of W and Co in both metallic and oxidised form, before and after pre-treatment (Figure 8). The peaks at 781 and 797 eV correspond to Co^2+^, whereas the peaks at 793 and 778 eV correspond to metallic Co; the peaks at 37.5 and 35.3 eV correspond to W^6+^, whereas 33.2 and 31.2 represent metallic W [25]. After acidic pre-treatment, the relative intensity of Co peaks decreases, whereas W peaks increase, confirming that exposure of the substrate to acidic solution dissolves Co quicker than W.

**Figure 9:**
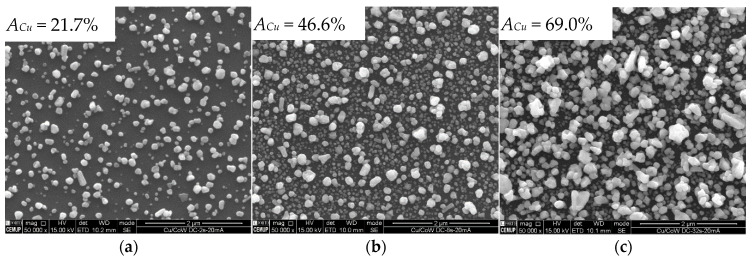
SEM images of Cu particles on Co-W substrate electrodeposited by DC at 20 mA·cm^−2^ for periods of 2 (**a**), 8 (**b**) and 32 s (**c**).

**Figure 10:**
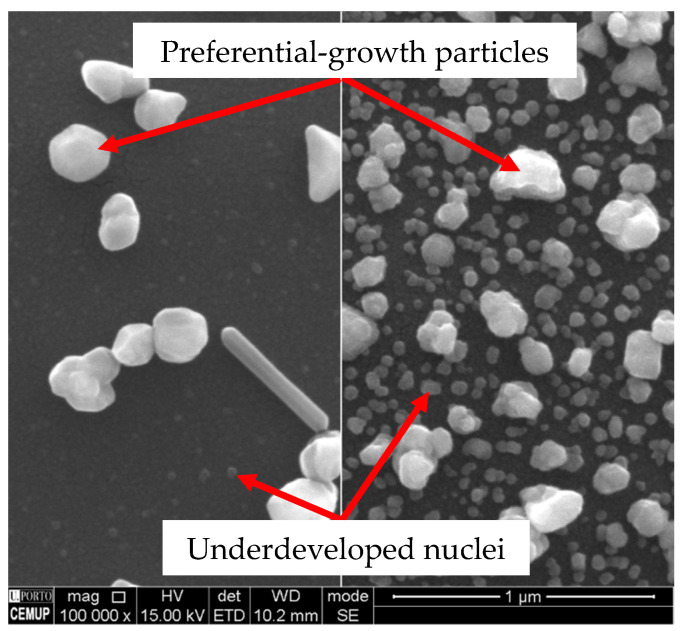
Nuclei density and size difference between PC and DC deposition for the same transferred charge, 160 mC.

**Figure 11:**
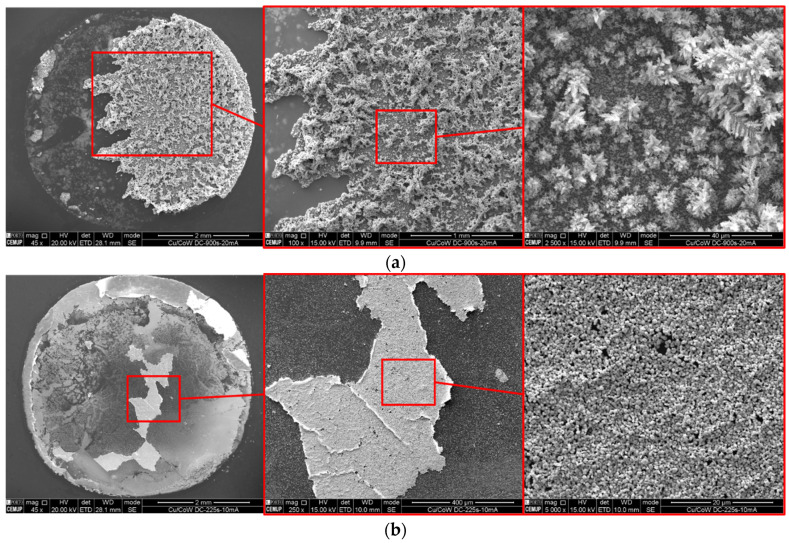
DC electrodeposited Cu on Co-W substrate at 20 (**a**) and 10 mA·cm^−2^ (**b**), for 900 and 225 s, respectively.

After 2 s of DC deposition, the nuclei density seems equivalent to that observed in PC after 52 s, which comprises the same amount of transferred charge according to Equation (1) (for DC, *η* = 1). The nuclei continue to increase in number and in size up to 8 s (DC), contrasting with what happens in PC for equivalent *Q*, where the number of nuclei and growing particles remain practically unchanged (Figure 9). The off time between pulses in PC deposition enhances the preferential growth of a few nuclei, resulting in a strong bimodal particle size (Figure 10). During *t_off_*, the current measured in the instrument is negligible and the corresponding cathodic potential applied is in the order of only a few mV. The dissolution rate is, thus, likely to be higher during *t_off_* than during *t_on_*, which may be controlling nuclei density by the dissolution of the less attached Cu particles or by dissolving the Co-W substrate directly underneath them. Another possible explanation for a lower nuclei density in PC deposition is that the number of active sites for nucleation decreases with the immersion time. It has been reported that the number of electrodeposited Cu nuclei decreases with increasing W content in Co-W and Ru-W substrates [15,26]. Accordingly, in this case, the selective dissolution of Co leads to substrate enrichment in W, rendering it difficult to nucleate further as the immersion time increases. In DC, there is no interval time and the total amount of charge is transferred continuously within only a few seconds. The average current density, *J*, which can be calculated dividing Equation (1) by *t*, is much higher in DC, *J* = 20 mA·cm^−2^, than in PC, *J* = 0.77 mA·cm^−2^. A similar *J* value was reported using a similar electrolyte in DC mode (1 mA·cm^−2^) [15], resulting in incomplete substrate coverage, similar to Figure 5a, suggesting that higher values of *J* are required to achieve better substrate coverage. Exposure time to the electrolyte is also substantially shorter in DC than in PC for the same transferred charge, reducing the extent of nuclei and substrate dissolution. Therefore, DC facilitates a denser nucleation and weakens the development of preferential orientation, leading to a more uniform growth of the nuclei (Figure 11). Longer DC deposition times of up to 32 s do not seem to increase the nuclei density but result mainly in particle growth, especially in the direction perpendicular to the substrate. Although substrate coverage improves, the method used for measuring this coverage fails to give an accurate result in this particular case. Higher values of *A_Cu_* obtained after 32 s of DC deposition are largely due to the protuberant particles that grow outwards and overlap the substrate, without effectively covering it. These substrate areas left uncovered will eventually undergo complete dissolution, whereas further particle growth will result in a discontinuous branched-like structure, mechanically inconsistent and poorly adhered to the substrate, as can be seen after 900 s of deposition in Figure 11a. Lowering the current density to 10 mA·cm^−2^ yields a similar result after 225 s of deposition. Although handled with care, these films are easily torn apart after deposition during rinsing and drying. The film consists of a discontinuous aggregate of Cu particles that detaches from the substrate (Figure 11b).

**Figure 12:**
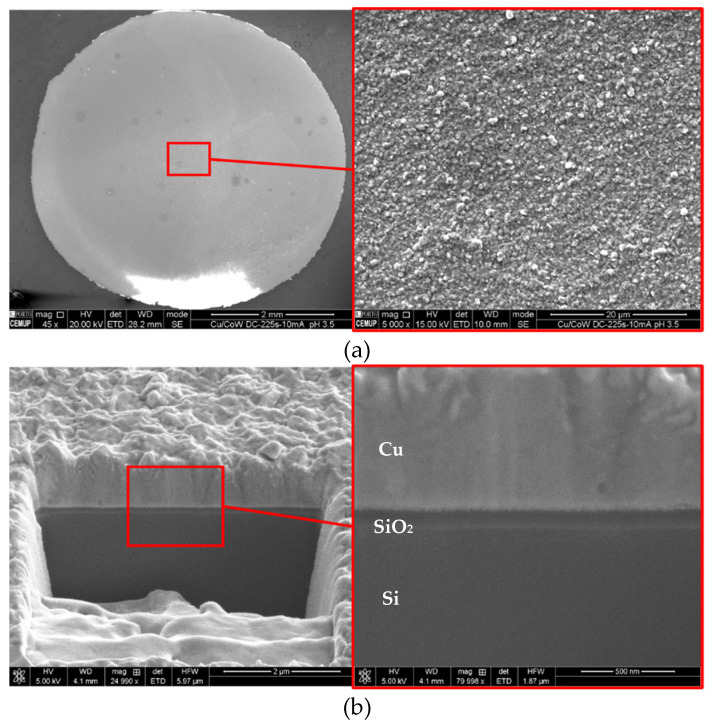
Film surface (**a**) and cross-section (**b**) of DC electrodeposited Cu on Co-W substrate at 10 mA·cm^−2^ for 225 s, in modified electrolyte (pH 3.5).

A method that could quickly coat the substrate with Cu is required to isolate the Co-W layer from the electrolyte and prevent its dissolution, which cannot be achieved by simply increasing the current density, since hydrogen reduction at the solid–liquid interface becomes a major problem, even with stirring. Although hydrogen bubbling was not observed during the first few seconds of deposition even at current densities higher than 5 mA·cm^−2^, for longer times small bubbles were seen forming at the border of the substrate, reducing the overall deposition efficiency. Alternatively, a decrease in substrate dissolution rate would likely allow a higher nucleation and a more uniform particle growth, resulting in improved substrate coverage. Therefore, we prepared a less aggressive version of the electrolyte by pouring a portion of the original electrolyte (pH 1.8) in a beaker, progressively adding a few drops of concentrated NaOH solution until pH 3.5 was reached. The addition of NaOH readily precipitates copper hydroxide that dissolves by stirring the solution for approximately 30 min. The modified electrolyte was used to electrodeposit Cu films with a DC density of 10 mA·cm^−2^ with very different results. The outcome is a compact surface structure, covering the whole substrate area, as depicted in Figure 12a. Cross-section imaging reveals a dense Cu film with thickness around 0.8–0.9 µm, with an average deposition rate of 3.5–4 nm/s and structural continuity across the Cu/Co-W/SiO_2_/Si interfaces (Figure 12b). Using Equation (5), a theoretical value of thickness, h, can be calculated, where M and ρ are the molecular mass and the density of Cu, respectively; e is the charge of the electron; O is the oxidation state for Cu ions; and N Avogadro’s number. The value of h obtained with 225 s of deposition at 10 mA·cm^−2^ is 0.84 µm, corresponding very closely to the measured Cu cross-sectional thickness, indicating a deposition efficiency near 100%.

(5) $$h=\frac{JMt}{\rho eON}$$

Although the Co-W film thickness is not possible to resolve in SEM, the region between SiO_2_ and Cu displays a continuous morphology, suggesting a good Cu film adhesion to Co-W. Evidently, higher resolution imaging is required to fully characterise the Cu/Co-W interface, supplemented by adhesion tests to evaluate its strength. Nevertheless, it is very clear that an increase in electrolyte pH from 1.8 to 3.5 yields a remarkable improvement in substrate coverage and film morphology. Substrate dissolution is likely to be much slower in pH 3.5, resulting in a slower decrease in the number of active sites for nucleation due to W enrichment, allowing a higher nuclei density to be formed and a more homogeneous particle growth. A prior study [24] demonstrated that a continuous Cu film can be effectively deposited onto Co using a neutral pH electrolyte containing copper sulphate and potassium sodium tartrate, where the dissolution of Co substrate is mitigated. However, the authors used a carbon-based activation pre-treatment on the Co surface to enhance deposition. In the present study, an increase in pH from 1.8 to 3.5 seems to be sufficient to prevent significant substrate dissolution, allowing a complete coverage and a sound interface. It is a much simpler and less disruptive approach from the current Cu interconnect technology standpoint.

## 4. Conclusions

Seedless Cu electroplating was performed on top of near equimolar Co-W thin films, as candidates for diffusion barrier layers for advanced Cu interconnect metallisation, using conventional copper sulphate acidic electrolytes of pH 1.8 and a modified version with pH 3.5. The main conclusions derived from this study are:

- The two main factors affecting Co-W substrate coverage and Cu film morphology are (1) the average current density and (2) the electrolyte aggressiveness to the substrate (indirectly, substrate corrosion rate).
- An adequate balance between these two factors is key for achieving good substrate coverage and Cu film compactness.
- Differences observed between pulsed- and direct-current modes are due to the fact that effective nucleation density is higher in the latter, where substrate dissolution is less extensive.
- The number of active sites for nucleation decreases over time as the substrate is exposed to the electrolyte, dissolving Co quicker than W, rendering incomplete/discontinuous substrate coverage with Cu.
- A less aggressive/acidic electrolyte with pH 3.5 is successful in slowing down substrate dissolution, yielding a dense direct-current electrodeposited Cu film displaying interfacial continuity with the substrate.

## Data Availability

The data presented in this study are available on request from the corresponding author.
